# Sacred Trees

**Published:** 1890-12

**Authors:** 


### SACRED TREES.

The palm, the oak, and the ash are the three trees which, since time immemorial, were held to be sacred trees. The first among them, which figures on the oldest monuments and pictures of the Egyptians and Assyrians, is the date palm, which was the symbol of the world and of creation, and the fruit of which filled the faithful with divine strength, and prepared them for the pleasures of immortality. The Jews and the Arabs again looked upon the same tree as a mystical allegory of human beings, for, [like them, it dies when its head (the summit) is cut off, and when a limb (branch) is once cut off it does not grow again. When Winifred of Devonshire (680–754 A. D.) went forth on his wanderings through Germany to preach the Gospel, one of his first actions was to cut down the giant oak in Saxony, which was dedicated to Thor and worshipped by the people far and near. In the abbey of Vetrou in Brittany, stood an oak tree which had grown out of the staff of St. Martin, the first abbot of the monastery, and in the shade of which the princes of Brittany prayed whenever they went into the abbey. The Celts, Germans and Scandinavians worshipped the mountain ash, to whom it was the holiest among trees.

